# The Landmark Series: Cancer Vaccines for Solid Tumors

**DOI:** 10.1245/s10434-024-16712-9

**Published:** 2024-12-20

**Authors:** Emily K. Ninmer, Feifan Xu, Craig L. Slingluff

**Affiliations:** 1 Department of Surgery/Division of Surgical Oncology and the Human Immune Therapy Center, Cancer Center, University of Virginia, Charlottesville, VA USA; 2 Department of Pathology, University of Virginia, Charlottesville, VA USA; 3 School of Medicine, Cancer Center, University of Virginia, Charlottesville, VA USA

**Keywords:** Cancer vaccine, Shared antigens, Neoantigens, Immunotherapy

## Abstract

Immunotherapy has become an integral part of the treatment for solid tumors. Cancer vaccines represent a potentially powerful class of immunotherapeutic agents to drive antitumor immunity. Cancer vaccine development involves selecting immunogenic target antigens expressed by tumor cells that can be effectively delivered for uptake by antigen-presenting cells to generate a robust adaptive immune response against tumor. While numerous cancer vaccines have been shown to produce antigen-specific immune responses, translating promising results of immunogenicity from early-phase trials into durable clinical benefit in larger randomized trials has remained elusive. Recent findings support new enthusiasm for several cancer vaccine approaches for solid tumors. This review will discuss landmark historic clinical trials in cancer vaccine development and strategies to optimize cancer vaccines to achieve improved clinical efficacy.

Immunotherapy has emerged as an important treatment of a range of solid tumors, typically with a more favorable safety profile than conventional therapies and with the potential to provide durable clinical benefit beyond initial treatment. Vaccines hold promise as cancer immunotherapy given their ability to stimulate an antigen-specific immune response ideally targeted to induce tumor regression, eradicate residual disease, and generate a memory immune response. Early cancer vaccine studies produced initial enthusiasm with evidence of robust antitumor responses after vaccination, but their clinical efficacy has been disappointing.^1^ A greater understanding of the complex mechanisms involved in tumor immunity has fostered renewed interest in cancer vaccines. This review will discuss challenges in improving clinical efficacy of cancer vaccines by highlighting landmark trials in cancer vaccine development. These trials are summarized in Table 1 (randomized phase III trials) and Table 2 (early-phase trials).

**Table 1:** Landmark randomized phase III cancer vaccine trials for solid tumors

Vaccine trial	Year	Antigens	Study population	Comparison	Clinical efficacy^a,b^
SWOG-9035 trial (Melacine with DETOX)^5–8^	2002	Tumor cell (allogenic)	Resected stage II melanoma (*n* = 689)	Observation	Intention-to-treat population: RFS HR 0.86 (95% CI 0.70–1.07); *p* = 0.18 OS HR 0.88 (95% CI 0.69–1.13); *p* = 0.31 HLA subgroups: HLA-A2^+^ and/or HLA-Cw3^+^ RFS HR 0.67 (99% CI 0.43–1.04); *p* = 0.02 OS HR 0.62 (99% CI 0.37–1.02); *p* = 0.01
MMAIT-IV trial (Canvaxin with BCG adjuvant) [NCT00052156]^10^	2017	Tumor cell (allogenic irradiated)	Stage IV melanoma with resection of up to 5 distant metastases (*n* = 496)	Placebo + BCG	DFS HR 0.88 (95% CI 0.71–1.10); *p* = 0.260 OS HR 1.04 (95% CI 0.80–1.35); *p* = 0.773 10-year OS rates (vaccine vs. placebo + BCG): 33.0% vs. 36.6
MAVIS trial (seviprotimut-L with alum adjuvant ) [NCT01546571]^14^	2021	Shed antigens	Resected stage IIB-III melanoma (*n* = 347)	Placebo	Intention-to-treat population: RFS HR 0.88 (95% CI 0.63–1.23); *p* = 0.46 OS HR 0.64 (95% CI: 0.34–1.18); *p* = 0.15 Stage IIB/C and <60 years subgroup: RFS HR 0.324 (95% CI 0.121–0.864)
gp100 peptide vaccine with IFA + systemic IL-2 [NCT00019682]^16^	2011	gp100_209-217(210M)_	Locally advanced stage III or stage IV melanoma without brain metastases (*n* = 185)	Systemic IL-2	Combination therapy versus monotherapy Overall response rate: 16% vs. 6% (*p* = 0.03) Median PFS: 2.2 versus 1.6 months (*p* = 0.008) Median OS: 17.8 versus 11.1 months (*p* = 0.06)
gp100 peptide vaccine with IFA + ipilimumab [NCT00094653]^17^	2010	gp100_209-217(210M),_ gp100_280-288(288V)_	Unresectable stage III or IV melanoma with disease progression on therapy for metastatic disease (*n* = 676)	Ipilimumab or gp100 vaccine monotherapy	Combination therapy vesus vaccine alone: OS HR 0.68 (95% CI 0.55–0.85); *p* < 0.001 Combination therapy versus ipilimumab alone: OS HR 1.04 (95% CI 0.83–1.30); *p* = 0.76 Ipilimumab alone versus vaccine alone: OS HR 0.66 (95% CI 0.51–0.87); *p* = 0.003
PRESENT trial (nelipepimut-S + GM-CSF) [NCT01479244]^19^	2019	HER2	Resected early stage, node-positive, low-to-intermediate HER2 expression (1+ or 2+ on IHC) females with breast cancer (*n* = 758)	Placebo	DFS HR 1.564 (95% CI 0.960–2.549); *p* = 0.07 Imaging detected recurrences (vaccine vs. placebo): 54.1% vs. 29.2% (*p* = 0.069)
DERMA trial (MAGE-A3 + AS15 adjuvant) [NCT00796445]^24^	2018	MAGE-A3	Resected stage III melanoma (*n* = 1345)	Placebo	DFS HR 1.01 (95% CI 0.88–1.17); *p* = 0.86
MAGRIT trial (MAGE-A3 + AS15 adjuvant) [NCT00480025]^25^	2016	MAGE-A3	Resected stage IB-IIIA nonsmall-cell lung cancer (MAGE-A3^+^) (*n* = 2312)	Placebo	DFS HR 1.02 (95% CI 0.89–1.18); *p* = 0.74
IMPACT trial (sipuleucel-T with GM-CSF linked to PAP pulsed with APCs) [NCT00065442]^31^	2010	PAP	Metastatic, castration-resistant prostate cancer (*n* = 512)	Placebo	OS HR 0.77 (95% CI 0.61–0.97); *p* = 0.02

*OS* overall survival; *RFS* recurrence-free survival; *DFS* disease-free survival; *PFS* progression-free survival; *CI* confidence interval; *HLA* human leukocyte antigen; *BCG* bacillus Calmette-Guérin; *IFA* incomplete Freund’s adjuvant; *IL-2* interleukin-2; *GM-CSF* granulocyte-macrophage colony-stimulating factor; *HER2* human epidermal growth factor 2; *IHC* immunohistochemistry; *MAGE* melanoma antigen gene; *PAP* prostatic acid phosphatase; *APC* antigen-presenting cell

^a^Reference group is the comparison group unless otherwise specified

^b^Clinical outcomes are reported as hazard ratios (HR) if available

**Table 2:** Landmark early phase cancer vaccine trials for solid tumors

Vaccine trial	Year	Antigens	Study design	Study population	Clinical efficacy
Polyvalent, shed-antigen melanoma vaccine with alum adjuvant^13^	2001	Shed antigens	Double-blind, randomized phase II placebo-controlled trial	Resected stage III melanoma (*n*=38)	Vaccine versus placebo: Median RFS: 1.6 versus 0.6 years (*p* = 0.26) (Cox proportional hazards adjusted *p* = 0.04) Median OS: 4.8 versus 2.7 years (*p* = NS)
Mel44 trial with 12 class I and 6 class II MHC-restricted shared melanoma antigens (MELITAC 12.1, MELITAC 12.6) [NCT00118274]^34,35^	2024	Class I: gp100, tyrosinase, MAGE-A1, -A3, -A10, NY-ESO-1 Class II: gp100, tyrosinase, MAGE-A1, -A2, -A3, -A6	Multicenter, randomized phase II trial comparing MELITAC 12.6 to MELITAC12.1, with or without single, low-dose Cy given 5 days before vaccine	Resected stage IIB-IV melanoma (*n* = 167)	Reference group: MELITAC 12.1 ± Cy Landmark 2.5-yr OS in intention-to-treat population: HR 0.52 (95% CI 0.27–0.97); *p* = 0.04 Landmark 1-yr RFS in males (vs. females): HR 0.35 (95% CI 0.14–0.86); *p* = 0.02
Type 1 polarized dendritic cells pulsed with 2 class I and 6 class II MHC-restricted HER2 peptides [NCT02061332]^36^	2017	HER2	Single-center, randomized phase II trial comparing vaccine delivered by 1 of 3 routes: intralesional (IL), intranodal (IN), or both (ILN)	Females with biopsy-proven HER2^+^ DCIS or stage I IBC (*n* = 54)	Pathologic complete response in patients with anti-HER2 CD4^+^ immune response in sentinel lymph node: 28.6% (DCIS) versus 8.3% (IBC) (cumulative response *p* = 0.04)
Lipo-MERIT mRNA vaccine trial with melanoma TAAs (BNTIII) [NCT02410733]^37^	2020	NY-ESO-1 MAGE-A3 tyrosinase TPTE	Multicenter, dose-escalation, phase I trial with or without anti-PD1 therapy	Resected and unresected stage IIB-IV melanoma with expression of at least one vaccine TAA (*n* = 89)	Best objective response in 42 patients with measurable metastatic disease at enrollment: Vaccine monotherapy: 12% partial response; 28% stable disease Combination therapy: 35% partial response
Long peptide vaccine with up to 20 predicted personalized class I MHC-restricted neoantigens, admixed with poly-ICLC (NeoVax) [NCT01970358]^38^	2017	Predicted neoantigens	Single-center, phase I trial with or without anti-PD1 antibody	Stage III and IV melanoma (*n* = 6)	Median follow-up (25 months) No recurrence in 4 patients with stage IIIB/C disease Recurrence in 2 patients with stage IV disease followed by complete radiographic response anti-PD1 antibody therapy
KEYNOTE-942 mRNA vaccine trial with up to 34 predicted personalized neoantigens (mRNA-4157/V940) [NCT03897881]^40^	2024	Predicted neoantigens	Multicenter, randomized phase IIb trial of vaccine plus pembrolizumab therapy versus pembrolizumab monotherapy	Resected stage IIIB-IV melanoma	Reference group: pembrolizumab monotherapy RFS HR 0.561 (95% CI 0.309–1.1017); *p* = 0.053 18-month RFS: 79% versus 62% Distant metastasis free survival HR: 0.347 (95% CI 0.145–0.828); *p* = 0.013 24-month distant metastasis free survival: 92% versus 73%

*HR* hazard ratio; *OS* overall survival; *RFS* recurrence-free survival; *CI* confidence interval; *NS* not significant; *MHC* major histocompatibility complex; *TAA* tumor-associated antigens; *HER2* human epidermal growth factor 2; *NY-ESO* New York esophageal squamous cell carcinoma; *MAGE* melanoma antigen gene; *TPTE* transmembrane phosphatase with tensin homology; *Cy* cyclophosphamide; *poly-ICLC* polyinosinic-polycytidylic acid-poly-L-lysine carboxymethylcellulose; *DCIS* ductal carcinoma in situ; *IBC* inflammatory breast cancer; *mRNA* messenger ribonucleic acid; *PD1* programmed cell death protein 1

## Antigens

Most cancer vaccines are designed to induce T-cell responses to cancer antigens. These antigens can be classified as either tumor-associated antigens (TAAs) or tumor-specific antigens (TSAs). Tumor-associated antigens are nonmutated self-antigens, such as tissue differentiation antigens and cancer-testis antigens, which are present in some normal tissues but aberrantly expressed by tumor cells.^2^ Tumor-specific antigens are expressed exclusively by tumor cells and include neoantigens arising primarily from somatic genomic alterations.^3^

Cancer vaccines can target antigens that are either defined or undefined. Defined antigens can be further classified as shared or personalized. Defined shared antigens, including TAAs and some TSAs, are expressed in a subset of patients, whereas defined personalized antigens, primarily mutated neoantigens, are unique to the patient and may be predicted by using computational methods, with or without validation.^4^ Undefined antigen vaccines include a large number of antigens that are unclear before treatment, either isolated ex vivo from autologous or allogeneic whole tumor cells or lysates and loaded onto autologous antigen-presenting cells (APCs) or released at the tumor site after immunogenic cell death with subsequent recruitment of endogenous APCs.^4^

## Whole Tumor Cell-Based Vaccines

Many early cancer vaccines were cell-based and comprised of undefined antigens derived from autologous or allogeneic human cancer cell lines or tumor lysates either loaded on dendritic cells or administered in adjuvants to promote dendritic cell activation. These vaccines were designed to stimulate broad vaccine-specific CD8^+^ and CD4^+^ T-cell responses. Autologous tumor vaccines have the potential to induce responses to personalized mutated neoantigens, whereas allogeneic tumor vaccines may stimulate responses to numerous shared antigens, including nonmutated self-antigens and shared mutated neoantigens, without requiring identification before vaccine development. Melacine and Canvaxin were two early allogeneic tumor cell-based vaccines for melanoma.

Melacine was comprised of an allogeneic whole melanoma tumor cell lysate with detoxified Freund’s adjuvant (DETOX) tested as adjuvant therapy versus observation in patients with resected stage II melanoma in a randomized phase III trial. No significant survival benefit was found on primary analysis^5,6^; however, a preplanned subgroup analysis suggested significant benefit in overall survival (OS) and meaningful improvement in recurrence-free survival (RFS) within a subgroup defined by class I human leukocyte antigen (HLA) expression, specifically serologically defined HLA-A2 and HLA-Cw3 (Table 1).^7,8^ These results support the need for more studies of cancer vaccine efficacy in patient subsets defined by HLA expression.

Canvaxin was comprised of three irradiated whole-cell melanoma lines administered with bacillus Calmette-Guérin (BCG) as a vaccine adjuvant. Two large phase III randomized trials were performed in the adjuvant setting: one for patients with resected stage III melanoma, and one for patients with stage IV melanoma after resection of up to five distant metastases. In both trials, patients were randomized to receive either Canvaxin plus BCG or placebo plus BCG. No clinical benefit was observed from Canvaxin in either trial, and both trials were terminated early for futility after interim analyses.^9^ An extended follow-up protocol for the stage IV melanoma trial to obtain long-term survival data revealed impressive OS for patients on either arm, suggesting a potential benefit of aggressive surgical approaches in selected patients with advanced melanoma (Table 1).^10^

Challenges in translating promising immune response activity in early phase trials to durable clinical benefit for cell-based vaccines are not limited to Melacine and Canvaxin. A systematic review of 36 phase II and phase III cell-based cancer vaccine trials across multiple cancer types revealed largely negative studies.^11^ Limited efficacy of cell-based vaccines may be due to off-target effects arising from antigens shared on both tumor and normal cells, the presence of dominant antigens that promote tumor immune escape or limit T-cell responses to more relevant antigens, and suboptimal tumor cell pretreatment and selection of vaccine adjuvants that are not optimal for antigen processing and presentation.^12^

Another approach for a cell-derived vaccine used soluble antigen released into cell culture supernatants of several melanoma cell lines, which induced antibody and T-cell responses to antigens in the vaccines.^13^ The preparation was shown to include many known TAAs. A small randomized placebo-controlled phase II trial showed significant improvement in RFS in the vaccine arm after adjusting for risk factors for disease progression (Table 2),^13^ but this has not been validated in a larger definitive phase III trial. A modification of that vaccine (seviprotimut-L) was tested in a larger randomized placebo-controlled phase III trial, with no significant impact on RFS; however, there were promising findings in subgroup analyses that may warrant investigation of vaccine efficacy in patients with stage IIB-IIC melanoma and younger patients in a definitive phase III trial (Table 1).^14^

## Vaccines Targeting Defined Antigens

### Shared Nonmutated Tumor Antigens

Many vaccines targeting shared nonmutated antigens are peptide-based. Some recent vaccine formulations were developed by using proteins and nucleic acids, among other platforms, to test optimal antigen delivery. Tumor-associated antigens are presumed to be subject to central immune tolerance due to their expression on some normal cells; however, immunogenic TAAs have been identified for many cancers. Most early peptide vaccines used single epitopes derived from TAAs restricted by class I major histocompatibility complex (MHC) molecules to selectively elicit antigen-specific CD8^+^ T-cell responses.

Melanocyte differentiation proteins (e.g., gp100, tyrosinase) are melanoma TAAs that have been extensively studied. Early trials at the Surgery Branch of the National Cancer Institute using a class I MHC-restricted peptide, gp100_209–217(210M)_, induced highly levels of antigen-specific CD8^+^ T-cell responses but failed to prevent melanoma tumor recurrence.^15^ In a multicenter, randomized phase III trial, a vaccine comprised of this gp100 peptide emulsified in incomplete Freund’s adjuvant (IFA) plus systemic high-dose interleukin-2 (IL-2) versus high-dose IL-2 monotherapy was tested in patients with advanced melanoma. The reported outcomes favored the addition of the vaccine, with significantly improved overall clinical response rates and progression-free survival (PFS) and a strong trend to prolonged OS (Table 1).^16^ While these data supported clinical benefit of vaccine targeting a TAA, results from another trial testing combination immune checkpoint inhibitor (ICI) therapy with a similar gp100 vaccine did not demonstrate a clinical benefit from the addition of vaccine. In that randomized phase III trial, the clinical response to ICI therapy with or without a vaccine comprised of two modified class I MHC-restricted peptides, gp100_209–217(210M)_ and gp100_280–288(288V)_, was evaluated in patients with metastatic melanoma. The study showed improved OS with ICI monotherapy compared with the vaccine and no benefit with combination therapy compared to ICI monotherapy (Table 1).^17^ This lack of benefit when combined with ICI therapy reduced enthusiasm for vaccines targeting TAAs; however, this trial used a vaccine against only two class I MHC-restricted peptides derived from gp100 without enhancing tumor-cognate CD4^+^ T-cell responses. The favorable outcomes seen in the trial using a similar vaccine with IL-2 coadministration may have overcome the need for IL-2 production by CD4^+^ T cells to support activation and proliferation of antigen-specific T cells.

Human epidermal growth factor receptor 2 (HER2) is a TAA that is overexpressed in breast cancer and some other solid tumors. Nelipepimut-S is a class I MHC-restricted peptide derived from HER2 that was safe and immunogenic with promising clinical outcomes in early-phase vaccine trials.^18^ In a large randomized phase III trial, vaccination with a nelipepimut-S plus granulocyte-macrophage colony-stimulating factor (GM-CSF) as vaccine adjuvant compared with GM-CSF alone for patients with resected high-risk breast cancer showed no differences in DFS at prespecified interim analysis (Table 1).^19^ This trial was terminated early for futility after the interim analysis revealed a disproportionate number of imaging-detected recurrences in vaccinated patients.^19^ Imaging was obtained according to protocol requirements rather than for clinical indication, and imaging-detected recurrence did not require biopsy confirmation. These findings highlight important considerations in defining clinical endpoints for vaccine trials.

Cancer-testis antigens, including New York esophageal squamous cell carcinoma 1 (NY-ESO-1) and the melanoma antigen gene (MAGE) protein family, are TAAs that are expressed in male germline cells and placenta but also in a variety of cancers. While early phase trials of vaccines targeting class I MHC-restricted peptides derived from these antigens demonstrated immunogenicity,^20,21^ approaches to enhance immune response rates have included using whole proteins to elicit responses against multiple class I and class II MHC-restricted epitopes. In a phase II trial, immunogenicity and favorable clinical outcomes were shown with a vaccine targeting the MAGE-A3 protein with AS15 adjuvant (toll-like receptor (TLR) 4 and TLR9 agonists plus QS-21) in patients with MAGE-A3^+^ advanced melanoma.^22^ Similarly, a randomized, placebo-controlled phase II trial for patients with resected MAGE-A3^+^ stage IB-II nonsmall-cell lung cancer (NSCLC) demonstrated immunogenicity of the MAGE-A3 protein vaccine with a TLR4 agonist plus QS-21 (AS02B), although no significant differences in clinical outcome were observed.^23^ Subsequent large, randomized phase III trials testing the MAGE-A3 protein vaccine with AS15 adjuvant for resected melanoma and NSCLC were negative, with no benefit in disease-free survival (DFS) (Table 1).^24,25^ For melanoma patients, the low magnitude and frequency of CD8^+^ T-cell responses seen in the phase II trial may partially explain the negative impact seen in the phase III trial. While for patients with NSCLC, neither the phase II or III trial was able to link the robust humoral responses induced by vaccine to clinical outcome. Prior studies in melanoma have shown heterogenous intratumoral expression of TAAs, including MAGE-A3.^26,27^ Thus, vaccines targeting a single antigen may promote cancer immunoediting processes, such as antigen loss, that facilitate immune escape.^28,29^

Another approach to targeting TAAs involves loading APCs, typically dendritic cells, with defined antigens. One notable example is sipuleucel-T, the autologous APC-based vaccine loaded with a fusion protein comprised of a prostate tissue differentiation antigen, prostatic acid phosphatase (PAP), linked to GM-CSF. Sipuleucel-T is delivered to the patient in a series of intravenous infusions. Two multicenter, randomized, placebo-controlled phase III trials demonstrated significant prolongation of OS with sipuleucel-T vaccination in men with metastatic castration-resistant prostate cancer, leading to Food and Drug Administration (FDA) approval of the vaccine for this indication (Table 1).^30,31^ Sipuleucel-T is the only FDA-approved cancer vaccine for solid tumors, excluding preventative vaccines against oncogenic hepatitis B virus (HBV) and human papillomavirus (HPV), suggesting that targeting tissue differentiated antigens may hold promise in future vaccine designs despite other negative larger trials testing shared nonmutated antigen vaccines.

Studies to further understand antitumor immunity have demonstrated a critical role of tumor-cognate CD4^+^ T cells in promoting antigen-specific CD8^+^ T-cell responses by enhancing dendritic cell maturation and function, as well as by mediating direct tumor killing independent of CD8^+^ T cells and mobilizing other innate immune cells in the antitumor response.^32,33^ Thus, the limited efficacy observed in early shared antigen vaccines based on epitopes for CD8^+^ T cells may be partially explained by lack of tumor-cognate CD4^+^ T-cell responses to orchestrate an optimal antitumor response, but this had not been addressed directly until recently. Some class II MHC-restricted peptides for shared nonmutated antigens have been recognized and tested in early phase trials. In a multicenter, phase II trial, patients with resected high-risk melanoma were randomized to receive either a multipeptide vaccine of 12 class I MHC-restricted peptides (12MP) derived from TAAs plus six melanoma-specific class II MHC-restricted peptides (6MHP) or a vaccine of 12MP plus tetanus toxoid peptide, with or without a single low-dose of cyclophosphamide 5 days before vaccination, to assess the impact of tumor-cognate versus nonspecific CD4^+^ T-cell help on vaccine immunogenicity and efficacy.^34^ Initial results demonstrated immunogenicity of both vaccines but showed no significant survival differences between study arms^34^; however, post-hoc analysis of long-term clinical outcomes revealed significantly improved OS and a favorable trend to improved RFS with melanoma-cognate helper peptide vaccination, with greatest benefit in males (Table 2).^35^ These results provide support for designing a randomized phase III trial with melanoma helper peptide vaccination powered to detect differences in clinical outcome and highlights the need to study the impact of biologic sex on outcomes in vaccine trials.

Other approaches for inducing tumor-cognate CD4^+^ T cells have been explored. In a phase II trial, women with biopsy-proven HER2-positive ductal carcinoma in situ (DCIS) and stage I invasive breast cancer (IBC) were randomized to vaccination by one of three routes (intralesional, intranodal, or both) with type I polarized dendritic cells pulsed with two class I MHC-restricted and six class II MHC-restricted HER2 peptides prior to definitive surgical resection.^36^ Similar immune response rates were observed for all vaccination routes, and pathologic complete responses were observed in 29% of DCIS patients and 8% of IBC patients (Table 2).^36^ This study raises the possibility that vaccination against HER2 may be therapeutic for early-stage breast cancers with induction of antigen-specific CD8^+^ and CD4^+^ T-cell responses. Notably, the clinical response for DCIS patients correlated with immune response in the sentinel lymph node, but not peripheral blood, with a significantly increased vaccine-induced CD4^+^ Th1 response in the sentinel lymph node and trend to lower CD4^+^ and CD8^+^ T-cell responses in the peripheral blood.^36^ These results suggest the need for further investigation into the impact of neoadjuvant vaccination strategies on T-cell activation within intact regional lymph nodes to enhance the antitumor response and improve clinical efficacy of cancer vaccines.

Another approach to enhance antigen presentation after immunization involved a nanoparticulate liposomal mRNA vaccine with sequences encoding for four class I MHC-restricted TAAs tagged with tetanus toxoid epitopes and targeting signal sequences for optimal delivery to immature dendritic cells in the lymphoid tissue.^37^ In a phase I trial, patients with resected and unresected advanced melanoma who expressed at least one of the encoded TAAs received intravenous injection of vaccine; some cohorts also received ICI therapy. Results showed CD4^+^ T-cell–dominant responses and effective ex vivo tumor killing, as well as long-lived tumor-specific memory T-cell formation and a durable clinical response in some patients (Table 2).^37^ These data support further investigation into targeting shared nonmutated antigens with vaccines optimized to overcome central immune tolerance and induce strong tumor-specific CD4^+^ and CD8^+^ T-cell responses for improved clinical efficacy.

### Neoantigen-Based Cancer Vaccines

Neoantigens have been increasingly studied as potential targets for vaccines given that they are tumor-specific and not subject to central immune tolerance. Most studies of tumor neoantigens have emphasized the goal of personalized vaccines. Patient-specific neoantigens are predicted by computational methods with most identified as epitopes for CD8^+^ T cells, although some epitopes have been identified for CD4^+^ T cells.

Early-phase trials of personalized neoantigen vaccines have shown promising results. In six patients with resected high-risk melanoma, a long peptide-based vaccine comprised of up to 20 personalized neoantigens predicted to bind autologous HLA-A or HLA-B (class I MHC) admixed with TLR3 agonist (poly-ICLC) induced strong de novo CD4^+^ and CD8^+^ T-cell responses against 60% and 16% of the neoantigens, respectively, as well as vaccine-induced memory cell formation in all patients and favorable clinical outcomes (Table 2).^38^ Similar immune responses using this vaccination strategy were seen in patients with unresectable metastatic melanoma, NSCLC, and bladder cancer, with evidence of major pathologic response postvaccination in melanoma patients treated concurrently with ICI therapy.^39^ In a randomized, phase IIb trial comparing a lipid nanoparticle mRNA-based vaccine encoding up to 34 predicted personalized neoantigens in combination with ICI therapy versus ICI monotherapy in patients with resected high-risk melanoma, meaningful prolongation in RFS was found in the vaccine-treated group compared with ICI monotherapy with minimal treatment-related toxicity (Table 2).^40^ These results have prompted a large definitive phase III randomized trial with this vaccine that is currently underway (NCT03897881).

Vaccines targeting shared neoantigens, typically representing driver mutations expressed by all cells within a patient’s tumor, have emerged as a scalable, cost-effective alternative to personalized neoantigen vaccines. In a phase I trial, a heterologous chimpanzee adenovirus and self-amplifying mRNA-based vaccine targeting 20 class I MHC-restricted validated shared neoantigens derived from common oncogenic driver mutations was given in combination with ICI therapy to patients with metastatic solid tumors expressing one of the encoded HLA-matched neoantigens.^41^ Some patients experienced stable disease after vaccination, but no objective responses were observed. Almost all patients expressed KRAS mutations; however, the results demonstrated reduced T-cell response to vaccine-encoded, tumor-relevant KRAS neoantigens in favor of vaccine-encoded, tumor-irrelevant TP53-specific T-cell responses, suggesting a need to enhance antigen presentation.^41^ These results highlight important considerations for the development of future multiepitope shared neoantigen vaccines that may impact clinical efficacy.

### Oncolytic Viruses

Oncolytic viruses are a new type of immunotherapy mimicking in situ vaccination using a viral vector that selectively infects and lyses tumor cells with release of tumor antigens to promote an antitumor immune response. Talimogene laherparepvec (T-VEC) is an oncolytic virus modified from the herpes simplex virus 1 with additional modification to enhance GM-CSF production that is injected directly into solid tumors. A randomized phase III trial of T-VEC versus GM-CSF demonstrated improved durable response rates for at least 6 months in patients with unresectable melanoma, leading to FDA approval of T-VEC for regional metastases of melanoma.^42^ T-VEC has been shown to produce abscopal effects, suggesting the promotion of systemic antitumor immunity arising from intralesional injection.^43^ Given the ability of intratumoral therapies to directly alter the tumor microenvironment and promote in situ vaccination effects, leveraging oncolytic viruses in combination with other treatment modalities may hold promise, but requires further investigation.^44^

### Prospects for Cancer Prevention with Vaccines

Vaccines against pathogens are effective at preventing infections. Similarly, the use of vaccines as a cancer prevention strategy for solid tumors in high-risk individuals may be an important clinical setting to optimize vaccine efficacy. Vaccines that prevent infections with the oncogenic viruses HBV and HPV have been successful in preventing hepatoma and HPV-associated cancers.^45,46^ Utilizing vaccines for cancer prevention is currently being explored in early-phase studies, including vaccines targeting KRAS mutations common in premalignant pancreatic lesions for patients at high risk for developing pancreatic cancer (NCT05013216) and TAAs for patients with Lynch syndrome at high risk for developing colon cancer (NCT05419011).

### Host Factors and Biomarkers for Cancer Vaccines

The immune response to vaccines is modulated by host factors, including sex, age, and the gut microbiome.^47–49^ The impact of these factors on outcomes after cancer vaccination has not been well characterized, although results from early phase trials suggest that this warrants further investigation to identify patients who may benefit most from cancer vaccines.^14,35^ The complexity of the immune response to vaccines presents challenges for identifying the measures of vaccine-induced immunity that best predict outcome. No biomarkers for vaccine efficacy have been formally validated and this remains an area of active investigation.

## Summary

Advances in cancer vaccine development to optimize antigen selection and delivery have yielded promising results in early-phase clinical trials. Improved bioinformatics techniques for predicting tumor neoantigens has led to growing interest in the development of personalized vaccines, which now show great promise. A deeper understanding of antitumor immunity has revealed critical components, such as the activation of tumor-cognate CD4^+^ T cells, which is required to drive effective antigen presentation and promote memory T-cell formation. Several challenges remain to enhance therapeutic efficacy of vaccines. Further study of the tumor microenvironment is needed to better enhance the ability of vaccine-induced T cells to infiltrate tumor and maintain function. Additional studies to incorporate vaccines in the neoadjuvant setting and to determine combination approaches with ICI therapy and other standard of care treatments are needed to optimize use of cancer vaccines in the clinic.
